# Tolerance to oral anticancer agent treatment in older adults with cancer: a secondary analysis of data from electronic health records and a pilot study of patient-reported outcomes

**DOI:** 10.1186/s12885-022-10026-3

**Published:** 2022-09-03

**Authors:** Yun Jiang, Madilyn Mason, Youmin Cho, Ankita Chittiprolu, Xingyu Zhang, Karen Harden, Yang Gong, Marcelline R. Harris, Debra L. Barton

**Affiliations:** 1 University of Michigan School of Nursing, Ann Arbor, MI USA; 2 Department of Systems, Populations, and Leadership, University of Michigan School of Nursing, 400 North Ingalls Building, Room 4160, Ann Arbor, MI 48109 USA; 3 Thomas E. Starzl Transplantation Institute, University of Pittsburgh, Pittsburgh, PA USA; 4 The University of Texas Health Science Center at Houston School of Biomedical Informatics, Houston, TX USA

**Keywords:** Oral anticancer agents, Capecitabine, Older adults, Adverse effects, Dose reduction

## Abstract

**Background:**

More than 60% of cancer cases occur in older adults, and many are treated with oral anticancer agents. Yet, the treatment tolerability in older adults has not been fully understood due to their underrepresentation in oncology clinical trials, creating challenges for treatment decision-making and symptom management. The objective of this study was to investigate the tolerance of capecitabine, an example of oral chemotherapy, among older adults with cancer and explore factors associated with capecitabine-related side effects and treatment changes, to enhance supportive care.

**Methods:**

A secondary analysis used combined data from electronic health records and a pilot study of patient-reported outcomes, with a total of 97 adult patients taking capecitabine during 2016–2017, including older adult patients aged 65 years or older (*n* = 43). The data extracted included patient socio-demographics, capecitabine information, side effects, and capecitabine treatment changes (dose reductions and dose interruptions). Bivariate correlations, negative binomial regression, and multiple linear regression were conducted for data analysis.

**Results:**

Older adults were more likely to experience fatigue (86% vs. 51%, *p* = .001) and experienced more severe fatigue (*β* = 0.44, *p* = 0.03) and hand-foot syndrome (HFS) (*β* = 1.15, *p* = 0.004) than younger adults. The severity of fatigue and HFS were associated with the number of outpatient medications (*β* = 0.06, *p* = 0.006) and the duration of treatment (*β* = 0.50, *p* = 0.009), respectively. Correlations among side effects presented different patterns between younger and older adults. Although more older adults experienced dose reductions (21% vs. 13%) and dose interruptions (33% vs. 28%) than younger adults, the differences were not statistically different. Female sex, breast cancer diagnosis, capecitabine monotherapy, and severe HFS were found to be associated with dose reductions (*p-values* < 0.05).

**Conclusions:**

Older adults were less likely to tolerate capecitabine treatment and had different co-occurring side effects compared to younger adults. While dose reductions are common among older adults, age 65 years or older may not be an independent factor of treatment changes. Other socio-demographic and clinical factors may be more likely to be associated. Future studies can be conducted to further explore older adults’ tolerance to a variety of oral anticancer agents to generate more evidence to support optimal treatment decision-making and symptom management.

## Background

Oral anticancer agents (OAAs) have been used increasingly in cancer patient care and demonstrated significant effectiveness in the management of certain cancers [1]. Patients often prefer OAAs over intravenous (IV) chemotherapy because of the convenience and flexibility in administration [1]. However, like traditional IV chemotherapy, many OAAs have a low therapeutic index and narrow safety margins, which can pose a high risk of toxicities even within prescribed doses. OAA-related side effects often lead to patients’ intolerance of the treatment and cause OAA dose reduction or dose interruption (i.e., temporary treatment discontinuation), which can potentially interfere with optimal treatment effects. More than 60% of cancer cases occur in older adults who are 65 years and older [2]. It is also common for many older adults with cancer to have increased frailty and pre-existing comorbidities and take multiple concomitant drugs, making them at high risk for medication toxicities and less tolerant to cancer treatments [3]. However, these older adults are often underrepresented in oncology clinical trials that are conducted to investigate the occurrence of OAA-related side effects. Such underrepresentation can create a knowledge gap regarding older adults’ responses and tolerance to OAAs [4], which results in challenges for health care providers to appropriately prescribe OAAs and manage OAA-related toxicities.

Capecitabine, a commonly prescribed oral cytotoxic chemotherapy, is used to treat several types of cancers such as metastatic breast cancer and colorectal cancer. Various side effects have been reported with capecitabine treatments, such as hand-foot syndrome (HFS), diarrhea, nausea, fatigue, and mouth sores. Studies have noted increased incidences of severe capecitabine-related side effects in older adults [5, 6]. Yet, within current research, factors associated with the development of severe side effects of capecitabine among older adults have not been clearly identified or consistently reported. For example, Leicher et al. [7] identified no relationship between capecitabine dose and the incidence of HFS events, while Comella et al. [8] found that a 1000 mg/m^2^ twice daily dose was associated with lower rates of HFS than the dose of 1250 mg/m^2^ in older patients.

Patients under OAA treatments have extensive responsibilities and involvement in the self-management of their OAAs and related side effects with limited supervision from their healthcare providers [9]. As many severe side effects are difficult to self-manage by patients and their families at home, a greater understanding of the potential factors associated with the development of OAA toxicities in older adults can lead to more personalized OAA treatment plans and better support for side effect monitoring and management. Within the current clinical practice, older adults’ OAA treatment decisions are often made on a day-to-day basis by accounting for the individuals’ response and tolerance [10]. Such real-world practice data have been documented in electronic health records (EHRs), creating the opportunity for in-depth secondary data analysis, and generating real-world evidence to support a better understanding of older adults’ tolerance of OAA treatments [11]. Relevant patients’ personal and clinical information can also be extracted from EHRs and used for the further exploration of influential factors of patient experiences with OAA-related toxicities [11].

The OAA treatment changes, such as dose reduction, usually act as countermeasures to resolve the effects of OAA intolerance. They may function as prime indicators of serious OAA-related side effects and are thus usually well-documented within EHRs such as clinical notes. Dose interruption, particularly a temporary suspension of the treatment by the health care provider, is also commonly due to the concern of severe side effects. Patient-initiated dose interruption may be related to unintentional missingness or intentional requests for a temporary break per personal reasons such as vacation, under the approval of the provider. Any dose interruptions that health care providers are aware of are documented in EHRs and can be extracted for analysis. An examination of the severity of side effects of OAAs paired with the treatment changes of dose reductions and dose interruptions among these patients may reveal potential influential factors of OAA treatment tolerability and lay the groundwork for the determination of better treatment plans and supportive care for this understudied population. Therefore, the purpose of this study was to investigate the prevalence and severity of side effects of capecitabine, as an example of OAA, in older adults with cancer and explore factors associated with capecitabine-related side effects and treatment changes to enhance supportive care.

## Methods

### Study design and sample

This study was a combined secondary analysis of existing data from EHRs and a previous pilot study of patient-reported outcomes (PROs) [12]. Data extracted from EHRs were considered as a complement to self-reported data collected from patient participants in the pilot study. The combination of the two datasets was also expected to increase the power of statistical analysis. Among all 383 EHR patients who received the prescription of capecitabine in their medication orders from January 1 – December 31, 2016, 50 patients were randomly selected and their EHR data including clinical notes were extracted for analysis. Among the 50 patients selected, three patients were excluded as they did not start taking capecitabine due to limited health insurance coverage (*n* = 2) or being transferred to another hospital (*n *= 1). EHR data from the remaining 47 patients were included in this combined data analysis. The original pilot study was an observational, single-group study that explored the relationships between capecitabine adherence, side effects, and side effect self-management among 50 adult patients who were diagnosed with gastrointestinal (GI) cancers and had taken capecitabine for at least two cycles [12]. This secondary analysis only used the telephone-collected patient-reported side effect data from all 50 participants. The final combined dataset consisted of 97 adult patients who were taking capecitabine between 2016–2017. Following the initial data assessment, no overlap of patients from the two data sources was identified. This study was carried out in accordance with the ethical guidelines of the 1975 Declaration of Helsinki and was approved by the Institutional Review Boards of the University of Michigan Medical School (IRBMED).

### EHR data extraction and clinical notes annotation

All EHR data were extracted from a Research Data Warehouse (RDW) through the Data Office of Clinical and Translational Research at the University of Michigan. The unstructured clinical notes (free text data), such as, physician progress notes, emergency department notes, nursing notes, and telephone notes, of 47 patients were annotated by a team of two annotators trained to extract information related to patient clinical characteristics, capecitabine treatment details, side-effect experience, and occurrence of capecitabine-related treatment changes. Patient socio-demographic variables were primarily identified from structured medical records, including age, race, ethnicity, sex, and marital status. Patient clinical characteristics included cancer diagnosis, cancer stage, number of comorbidities, and number of outpatient medications. The capecitabine treatment details were comprised of capecitabine treatment start dates, daily dose, cycle pattern, and treatment type (monotherapy vs. in combination with other chemotherapy). To distinguish between cancer side effects and capecitabine-related side effects, the annotators referenced the medication side-effect database, SIDER, which contains all recorded potential side effects of marketed medications from public documents and package inserts [13]. For each identified capecitabine side effect, the name and highest severity experienced were extracted and coded from 1 = mild to 4 = very severe based on the description in the notes. The dates when the side effect occurred were also extracted. With patient clinical notes that did not explicitly state the severity of patients’ side effects, the annotators referenced the National Cancer Institute Common Terminology for Adverse Effects (NCI CTCAE) to map the description of side effects in the notes to the grade of adverse effects (grade 1–4) in the NCI CTCAE [14]. Occurrences of the treatment changes during the study period (January 1 to December 31, 2016), including dose reduction and dose interruption, were identified and classified as 0 = not present and 1 = present. Further coding was applied to manage both structured and unstructured data extracted from EHRs. For example, for the capecitabine cycle pattern, a 6-point coding system was assigned with 1 = continuous, 2 = 7 days on, 7 days off, 3 = 14 days on, 14 days off, 4 = 14 days on, 7 days off, 5 = 21 days on, 7 days off, and 6 = other. To address the interest of older adults’ tolerance to OAA treatments, patient age was additionally grouped by either under 65 years old vs. 65 years old or above.

### Pilot study patient-reported outcome data

The pilot study collected the severity of 8 common side effects of capecitabine directly from enrolled 50 patients using the Patient-Reported Outcomes version of Common Terminology for Adverse Effects (PRO-CTCAE) [15]. These side effects included fatigue, constipation, diarrhea, HFS, nausea, vomiting, mouth sores, and sleep difficulties. The severity of side effects was coded from 1 = mild to 4 = very severe. For patients without the experience of the side effect, the severity was coded as 0 = none. Patient socio-demographic information was collected using a short survey, including age, education, race, ethnicity, and marital status. Clinical characteristics such as patient cancer diagnosis, cancer stage, number of comorbidities, number of outpatient medications, treatment type, treatment intent, daily dose, cycle pattern, and occurrences of treatment changes (dose reduction and dose interruption) were extracted from patients’ medical records, using the same techniques and procedures as those mentioned above in EHR data extraction and clinical notes annotation. The details of patient recruitment and data collection have been reported elsewhere [12].

### Data analysis

Descriptive statistics were used to summarize variables, including patient socio-demographic characteristics, clinical factors, severity of common side effects of capecitabine, and treatment plan changes (i.e., dose reduction or dose interruption) during the study period. Correlation analysis, Chi-squared test or Fisher’s exact test, and Wilcoxon-Mann Whitney test or independent samples t-test were used to compare two sample characteristics, and the associations between socio-demographic, clinical characteristics, and the severity of each side effect and treatment changes. Specifically, for the severity of common toxicities, the differences in occurrences and mean severity between older (age $\ge$ 65) and younger (age < 65) adults were compared. To identify factors associated with the severity of fatigue (the side effect with high prevalence and normal distribution), we used multiple linear regression analysis. To identify factors associated with the severity of HFS (significantly experienced by older adults in bivariate analysis), we used negative binomial regression since the distribution of the severity of HFS was skewed to zero. Power analysis on multiple linear regression with 14 predictors, indicated that a sample size of 96 could obtain 0.80 power with an anticipated effect size of 0.22. A significance level of *p* < 0.05 was used for the regression models. Stata IC version 16.0 was used for the statistical analysis.

## Results

### Sample characteristics

As shown in Table 1, the mean age of all patients (*N* = 97) was 61.7 ± 12.3 years old, and 44.3% (*n* = 43) were older adults aged 65 years or above. Within all samples, most of the patients were male, white, diagnosed with advanced/metastatic cancer, and with pancreatic cancer as the most common cancer type. Most patients took capecitabine in combination with other chemotherapy, on the cycle of 14 days on and 7 days off, with an average initial daily dose of 2459 mg, having an average of 4 comorbidities, and having 10 outpatient medications prescribed. Compared to younger adults, older adult patients were more likely to be white (95.3% vs. 50.6%, *p* = 0.02), had a lower initial dose of capecitabine (2151 mg vs. 2709 mg, *p* = 0.009), and took significantly more outpatient medications (12 vs. 9, *p* = 0.01). Approximately 17% (*n* = 16) of patients experienced dose reductions during the study phase, and 30% (*n* = 29) experienced dose interruptions.

**Table 1:** Summary of sample characteristics by two data sources

	All Sample (N = 97)	Age < 65 years old (*n* = 54)	Age ≥ 65 years old (*n* = 43)	*p*	EHR Sample (*n* = 47)	Pilot Study Sample (n = 50)	*p*
	*n (%)*	*n (%)*	*n (%)*		*n (%)*	*n (%)*	
Age (years, Mean ± SD)	61.72 ± 12.26	–	–	–	59.55 ± 12.34	63.76 ± 11.95)	.09
65 years old	54 (55.67%)				30 (63.83%)	24 (48%)	.11
$\ge 65$ years old	43 (44.33%)				17 (36.17%)	26 (52%)	
Gender				.91			.05
Female	48 (49.48%)	27 (50.0%)	21 (48.8%)		28 (59.57%)	20 (40%)	
Male	49 (50.52%)	27 (50.0%)	22 (51.2%)		19 (40.43%)	30 (60%)	
Race				**.02**			.78
White	83 (85.57%)	42 (50.6%)	41 (95.3%)		39 (82.98%)	44 (88%)	
Non-White	14 (14.4%)	12 (22.2%)	2 (4.7%)		8 (17.02%)	6 (12%)	
Types of Cancer				.13			** < .001**
Breast	15 (15.46%)	9 (16.7%)	6 (14.0%)		15 (31.91%)	0 (0%)	
Colorectal	18 (18.56%)	14 (25.9%)	4 (9.3%)		9 (19.15%)	9 (18%)	
Pancreatic	40 (41.24%)	18 (33.3%)	22 (51.2%)		9 (19.15%)	31 (62%)	
Others	24 (24.74%)	13 (24.1%)	11 (25.6%)		14 (29.79%)	10 (20%)	
Stage of Cancer				.26			**.002**
Non-Advanced/metastatic	40 (41.24%)	25 (46.3%)	15 (34.9%)		12 (25.53%)	28 (56%)	
Advanced/metastatic	57 (58.76%)	29 (53.7%)	28 (65.1%)		35 (74.47%)	22 (44%)	
Treatment type				.52			**.04**
Monotherapy	28 (28.87%)	17 (31.5%)	11 (25.6%)		18(38.30%)	10 (20%)	
Combination therapy	69 (71.13%)	37 (68.5%)	32 (74.4%)		29 (61.70%)	40 (80%)	
Capecitabine daily dose (mg) (Mean ± SD)	2459.38 ± 1053.07	2709.4 ± 1053.24	2151.2 ± 979.2	**.009**	2621.74 ± 989.71	2310.00 ± 1096.79	.21
Days on treatment (days) (Mean ± SD)	49.76 ± 77.83	42.5 ± 60.6	58.9 ± 95.2	.33	52 ± 99.53	47.66 ± 50.60	**.003**
Cycle pattern				.24			** < .001**
Continuous	3 (3.12%)	1 (1.9%)	2 (4.7%)		0 (0%)	3 (6%)	
7 days on, 7 days off	9 (9.38%)	5 (9.3%)	4 (9.3%)		0 (0%)	9 (18%)	
14 days on, 14 days off	5 (5.21%)	5 (9.3%)	0 (0.0%)		3 (6.52%)	2 (4%)	
14 days on, 7 days off	62 (64.58%)	32 (59.3%)	30 (69.8%)		30 (65.22%)	32 (64%)	
21 days on, 7 days off	4 (4.17%)	1 (1.9%)	3 (7.0%)		0 (0%)	4 (8%)	
Others	7 (7.29%)	5 (9.3%)	2 (4.7%)		7 (15.22%)	0 (0%)	
Number of comorbidities (Mean ± SD)	3.90 ± 2.31, range 1–12	3.93 ± 2.12, range 1–12	3.86 ± 2.57, range 1–10	.89	5.40 ± 2.41, range 1–21	2.48 ± 0.89, range 1–4	** < .001**
Number of outpatient medications (Mean ± SD, range)	10.14 ± 5.19, range 1–28	9.02 ± 5.34, range 1–28	11.56 ± 4.67, range 2–23	**.01**	8.15 ± 5.34, range 1–28	12.02 ± 4.31, range 4–23	** < .001**
Nausea	44 (45.4%)	28 (51.9%)	16 (37.2%)	.15	22 (46.8%)	22 (44.0%)	.78
Vomiting	19 (19.6%)	14 (25.9%)	5 (11.6%)	.08	9 (19.1%)	10 (20.0%)	.92
Diarrhea	48 (49.5%)	26 (48.1%)	22 (51.2%)	.77	16 (34.0%)	32 (64.0%)	**.003**
HFS	29 (29.9%)	12 (22.2%)	17 (39.5%)	.06	11 (23.4%)	18 (36.0%)	.18
Fatigue	67 (69.1%)	30 (50.6%)	37 (86.0%)	**.001**	24 (51.1%)	43 (86.0%)	** < .001**
Constipation	34 (35.1%)	17 (31.5%)	17 (39.5%)	.41	9 (19.1%)	25 (50.0%)	**.001**
Mouth sores	19 (19.8%)	7 (13.0%)	12 (27.9%)	.07	6 (12.8%)	13 26.0%)	.10
Sleep difficulties	25 (25.8%)	15 (27.8%)	10 (23.3%)	.61	1 ((2.1%)	24 (48.0%)	** < .001**
Dose reduction (yes)	16 (16.49%)	7 (13.0%)	9 (20.9%)	.41	12 (25.53%)	4 (8%)	**.02**
Dose interruption (yes)	29 (29.90%)	15 (27.8%)	14 (32.6%)	.61	15 (31.91%)	14 (28%)	.67

Patients from two sets of samples were not significantly different in socio-demographic characteristics (i.e., age, sex, and race). However, there were statistically significant differences in clinical factors. The EHR sample included significantly more patients with advanced/metastatic stage of cancer (*p* = 0.002). They were on treatment for significantly longer days (*p* = 0.003), had a significantly higher number of comorbidities (*p* < 0.001), and were significantly more likely to have dose reductions (*p* = 0.02). Regarding the types of cancer, the PRO sample did not include patients with breast cancer but included significantly more patients with pancreatic cancer (*p* < 0.001). In both samples, most patients were on combination therapy rather than monotherapy, but the PRO sample had a significantly higher proportion on combination therapy (80% vs. 61.7%, *p* = 0.04). Although the proportion of different types of treatment cycles was significantly different in the two samples (*p* < 0.001), the most common cycle was 14 days on and 7 days off cycle in both samples. The PRO sample had a significantly higher number of outpatient medications (*p* < 0.001).

### Experience of common side effects

**Fig. 1:**
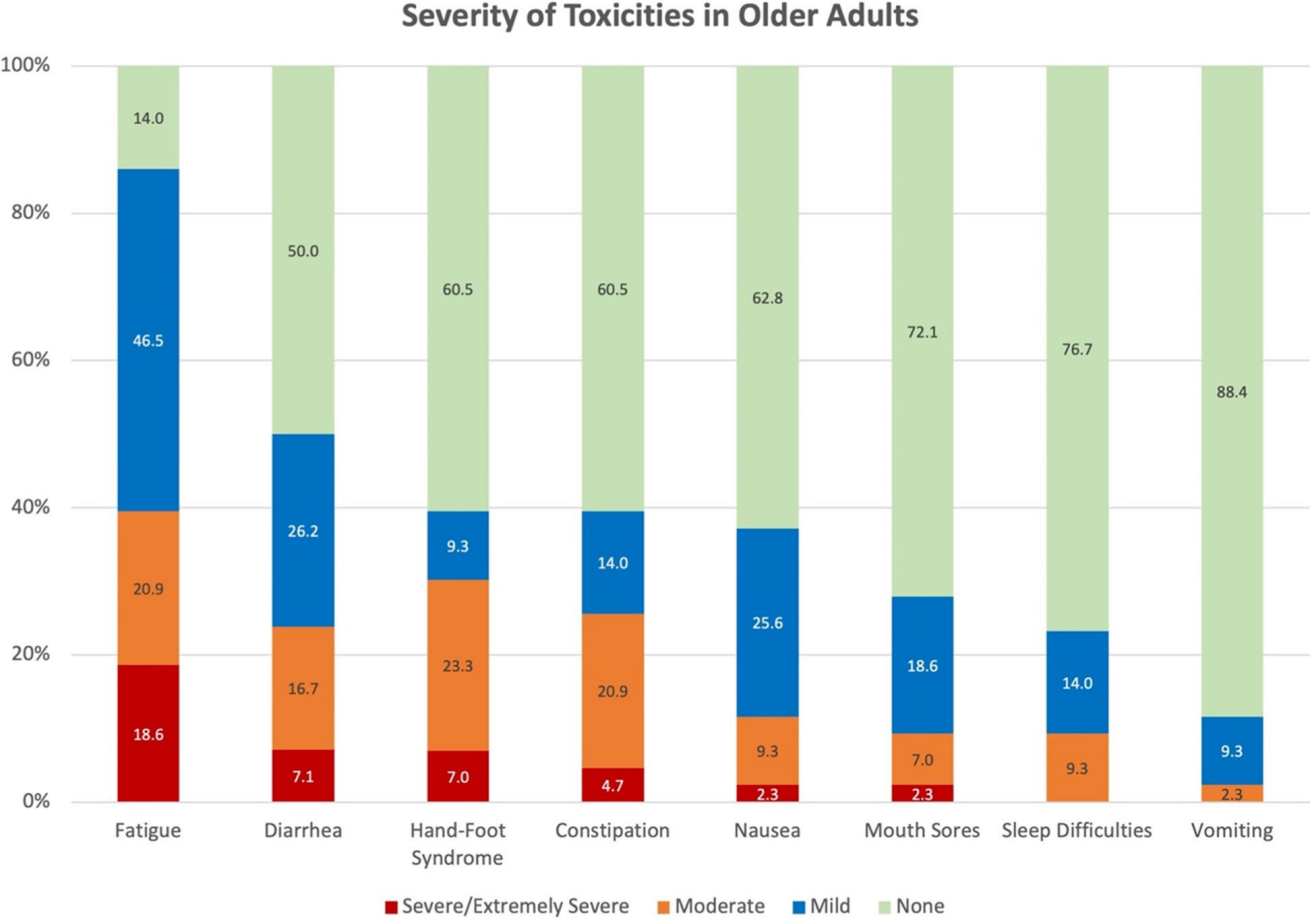
The severity of toxicities in older adults

Table 1 presents the frequency of eight common side effects of capecitabine. Although other types of side effects, such as taste change and hair loss, were also identified from EHR notes, these eight side effects were the top extractions, which matched with those from the pilot PRO study. Older adult patients were more likely to experience fatigue than younger adults (86.1% vs. 50.6%, *p* = 0.001), and they experienced more severe fatigue (*p* = 0.004) and HFS (*p* = 0.02) (see Table 2). The frequency and severity of the other six types of side effects were not significantly different between older and younger adults (*p-value*s > 0.05). Patients from the PRO sample were more likely to report severe diarrhea, constipation, fatigue, and sleep difficulties than those from the EHR sample (see Table 1 and 2). Figure 1 shows the level of severity of all eight side effects in older adults. Fatigue was rated as severe or extremely severe in 18.6% of older adults. Followed were severe/extremely severe diarrhea (7.1%) and HFS (7.0%). HFS was the most commonly experienced moderate side effect in older adult patients (23.3%), followed by fatigue (20.9%) and constipation (20.9%).

**Table 2:** The severity of capecitabine side effects by age groups and data sources

Severity of Side Effects	Age < 65 years old (*n* = 54)	Age $\ge 65$ years old (*n* = 43)	*p*	EHR Sample (*n* = 47)	Patient-Reported Outcome Sample (*n* = 50)	*p*
	Mean (SD)	Mean (SD)		Mean (SD)	Mean (SD)	
Nausea	0.81 (1.03)	0.51 (0.77)	.13	0.53 (0.62)	0.78 (1.04)	.56
Vomiting	0.43 (0.90)	0.14 (0.41)	.07	0.26 (0.61)	0.32 (0.77)	.88
Diarrhea	0.81 (1.07)	0.83 (1.03)	.86	0.50 (0.86)	1.08 (1.03)	**.002**
HFS	0.31 (0.72)	0.79 (1.10)	**.02**	0.40 (0.82)	0.60 (0.90)	.20
Fatigue	0.89 (0.96)	1.44 (0.96)	**.005**	0.72 (0.88)	1.52 (0.95)	** < .001**
Constipation	0.59 (1.06)	0.72 (1.03)	.40	0.26 (0.57)	0.96 (1.11)	** < .001**
Mouth sores	0.22 (0.66)	0.40 (0.73)	.08	0.21 (0.62)	0.38 (0.75)	.12
Sleep difficulties	0.43 (0.79)	0.33 (0.64)	.59	0.04 (0.29)	0.70 (0.86)	** < .001**

*HFS* Hand-Foot Syndrome

**Fig. 2:**
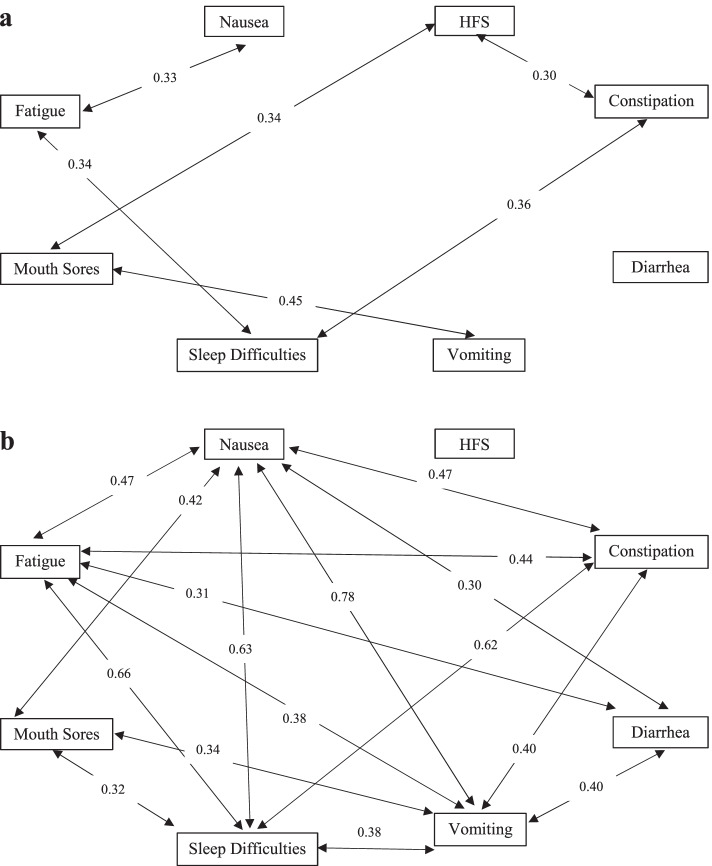
Correlation among toxicities in older adults (**a**) and younger adults (**b**). Note: *p*-value < 0.05

Figure 2 shows the correlations between the severity of side effects in older and younger adults. In older adult patients, the severity of fatigue was significantly correlated with the severity of nausea (*r* = 0.33), constipation (*r* = 0.35), and sleep difficulties (*r* = 0.33, *p-values* < 0.05). The severity of HFS was correlated with the severity of constipation (*r* = 0.30) and mouth sores (*r* = 0.34, respectively, *p-values* < 0.05). The severity of mouth sores was also correlated with the severity of vomiting (*r* = 0.44, *p* < 0.05). The severity of diarrhea was not correlated with any side effects. On the other hand, in younger adult patients, more correlations between side effects were identified. Specifically, nausea and vomiting were strongly correlated with each other (*r* = 0.78), and they were also correlated with all other side effects except HFS. The severity of sleep difficulties was moderately correlated with most other side effects, such as nausea (*r* = 0.63), fatigue (*r* = 0.66), and constipation (*r* = 0.62), except for diarrhea and HFS. HFS was not correlated with any other side effects in younger adult patients.

### Factors associated with severity of side effects

The regression model of the severity of fatigue and HFS is shown in Table 3. Specifically, after controlling for other variables in the models, older adult patients experienced significantly more severe fatigue (*β* = 0.44, *p* = 0.03) and HFS (*β* = 1.15, *p* = 0.004). The increased number of outpatient medications was also associated with more severe fatigue (*β* = 0.06, *p* = 0.006). Patients on capecitabine treatment for a longer time (more days) tended to report more severe HFS (*β* = 0.5, *p* = 0.009).

**Table 3:** Factors associated with the severity of HFS and fatigue

	HFS *p* = 0.02			Fatigue *F*(13,82) = 2.51, *p* = 0.006, *R-squared* = 0.28		
	*β*	*SE*	*p*	*β*	*SE*	*p*
Age $\ge 65$	1.15	0.40	**.004**	0.44	0.20	**.03**
Female	-0.07	0.43	.85	-0.45	0.22	.05
White	0.10	0.52	.83	-0.01	0.29	.97
Other cancer	Ref			Ref		
Breast Cancer	0.79	0.76	.30	0.11	0.39	.76
Pancreatic Cancer	0.57	0.56	.31	0.22	0.24	.36
Colorectal Cancer	1.10	0.60	.06	0.36	0.32	.26
Advanced/metastatic stage	0.11	0.40	.77	-0.34	0.20	.10
Initial daily dose (g)	0.30	0.21	.14	0.03	0.11	.78
Days on treatment	0.50	0.19	**.009**	0.08	0.09	.35
Combination treatment	-0.14	0.42	.73	-0.005	0.23	.98
14 days on, 7 days off cycle	-0.35	0.39	.35	-0.04	0.20	.82
Number of outpatient medications	-0.009	0.04	.81	0.06	0.02	**0.006**
Number of comorbidities	-3.90	0.09	0.42	0.008	0.04	.84

### Treatment plan changes

As shown in Table 4, more than half of the patients who experienced dose reductions were older adults (9/16, 56.3%). Although older adult patients tended to be more likely to experience dose reductions (21% vs. 13%) and dose interruptions (33% vs. 28%) than younger adult patients, the differences were not statistically significant. Dose reductions were more likely to occur in females (2-tailed Fisher’s exact test *p* = 0.006), patients diagnosed with breast cancer (2-tailed Fisher’s exact test *p* = 0.006), and patients on monotherapy of capecitabine (*p* = 0.04). Also, the mean severity of HFS was significantly higher in patients who experienced a dose reduction (*U* = 424.5, *p* = 0.007). Dose interruption was only found to be associated with the initial daily dose of capecitabine (*U* = 619, *p* = 0.003). The severity of eight common side effects was not significantly associated with capecitabine dose interruption.

**Table 4:** Bivariate analysis of associations between sample characteristics and treatment plan changes

	Dose Reduction			*p*	Dose interruption			*p*
	Yes (*n* = 16)	No (*n* = 81)			Yes (*n* = 29)	No (*n* = 68)		
	*n* (%)	*n* (%)			*n* (%)	*n* (%)		
Age (years old)			*X*^2^ (1, *N* = 97) = 1.10	.29			*X*^2^ (1, *N* = 97) = 0.26	.60
< 65 years old	7 (43.8%)	47 (58.0%)			15 (51.7%)	39 (57.4%)		
$\ge 65$ years old	9 (56.3%)	34 (42.0%)			14 (48.3%)	29 (42.6%)		
Gender			*2-tailed Fisher’s exact*	**.006**			*X*^2^ (1, *N* = 97) = 1.38	.24
Female	13 (81.3%)	35 (43.2%)			17 (58.6%)	31 (45.6%)		
Male	3 (18.8%)	46 (56.8%)			12 (41.4%)	37 (54.4%)		
Race			*2-tailed Fisher’s exact*	.45			*2-tailed Fisher’s exact*	1.00
White	15 (93.8%)	68 (84.0%)			25 (86.2%)	58 (85.3%)		
Non-White	1 (6.3%)	13 (16.0%)			4 (13.8%)	10 (14.7%)		
Types of Cancer			*2-tailed Fisher’s exact*	**.006**			*2-tailed Fisher’s exact*	.44
Breast	6 (37.5%)	9 (11.1%)			3 (10.3%)	12 (17.5%)		
Colorectal	5 (31.3%)	13 (16.0%)			6 (20.7%)	12 (17.5%)		
Pancreatic	2 (12.5%)	38 (46.9%)			15 (51.7%)	25 (36.8%)		
Others	3 (18.8%)	21 (25.9%)			5 (17.2%)	19 (27.9%)		
Stage of Cancer			*X*^2^ (1, *N* = 97) = 0.11	.74			*X*^2^ (1, *N* = 97) = 0.0003	.98
Non-advanced/metastatic	6 (37.5%)	34 (42.0%)			12 (41.4%)	28 (41.2%)		
Advanced/metastatic	10 (62.5%)	47 (58.0%)			17 (58.6%)	40 (58.8%)		
Treatment type			*X*^2^ (1, *N* = 97) = 4.16	**.04**			*X*^2^ (1, *N* = 97) = 0.03	.85
Monotherapy	8 (50.0%)	20 (24.7%)			8 (27.6%)	20 (29.4%)		
Combination therapy	8 (50.0%)	61 (75.3%)			21 (72.4%)	48 (70.6%)		
Capecitabine daily dose (mg) (Mean ± SD)	2906.25 ± 1128.70	2370.00 ± 1021.34	*U* = 464.5	.07	2051.72 ± 1175.23	2635.82 ± 951.67	*U* = 619	**.003**
Days on treatment (days) (Mean ± SD)	76 ± 150.49	44.58 ± 53.47	*U* = 539	.28	53.69 ± 104.92	48.09 ± 63.80	*U* = 940	.28
Cycle pattern			*2-tailed Fisher’s exact*	.40			*X*^2^ (1, *N* = 97) = 1.29	.25
14 days on, 7 days off	12 (75.0%)	50 (61.7%)			21 (39.7%)	41 (60.3%)		
Others	4 (25.0%)	31 (38.3%)			8 (27.6%)	27 (39.7%)		
Number of comorbidities (Mean ± SD)	4.44 ± 2.45	3.79 ± 2.28	*U* = 529	.23	4.24 ± 2.23	3.75 ± 2.35	*U* = 833	.22
Number of outpatient medications (Mean ± SD)	8.88 ± 5.52	10.40 ± 5.12	*t* (95) = 1.07	.28	10.45 ± 5.34	10.01 ± 5.16	*t* (95) = -0.37	.70
Nausea (severity) (Mean ± SD)	0.62 ± 0.72	0.69 ± 0.97	*U* = 633	.87	0.76 ± 1.06	0.65 ± 0.88	*U* = 932.5	.63
Vomiting (severity) (Mean ± SD)	0.19 ± 0.40	0.32 ± 0.79	*U* = 634	.84	0.48 ± 1.09	0.22 ± 0.51	*U* = 947.5	.66
Diarrhea (severity) (Mean ± SD)	0.67 ± 0.90	0.85 ± 1.07	*U* = 565	.64	1.07 ± 1.18	0.72 ± 0.97	*U* = 796.5	.17
Hand Foot Syndrome (severity) (Mean ± SD)	1.12 ± 1.26	0.41 ± 0.82	*U* = 424.5	**.007**	0.79 ± 1.24	0.41 ± 0.76	*U* = 848.5	.17
Fatigue (severity) (Mean ± SD)	1.00 ± 1.03	1.16 ± 0.99	*U* = 583	.50	1.34 ± 1.01	1.04 ± 0.98	*U* = 818.5	.16
Constipation (severity) (Mean ± SD)	0.44 ± 0.89	0.69 ± 1.07	*U* = 566.5	.35	0.59 ± 1.21	0.68 ± 0.97	*U* = 864	.25
Mouth sores (severity) (Mean ± SD)	0.44 ± 0.89	0.27 ± 0.65	*U* = 600.5	.50	0.41 ± 0.91	0.25 ± 0.58	*U* = 952.5	.70
Sleep difficulties (severity) (Mean ± SD)	0.19 ± 0.54	0.42 ± 0.76	*U* = 546.5	.19	0.38 ± 0.78	0.38 ± 0.71	*U* = 967.5	.84

## Discussion

As the utilization of OAAs for cancer management continues to rise, understanding OAA treatment tolerability in older adults is imperative to the development of personalized OAA treatment plans and symptom management strategies for this population [16]. This retrospective secondary analysis of combined EHR data with patient-reported outcome (PRO) data provided insights into side-effect experiences and treatment changes affecting older adult patients with cancer taking capecitabine. They were more likely to experience certain severe side effects such as fatigue and hand-foot syndromes than younger adults. The proportion of older adults who experienced dose reductions or interruptions was higher than that of younger adults, although the difference was not statistically significant. With the primary focus on older adults’ experiences of side effects, this study also identified potential factors associated with the severity of side effects of capecitabine and capecitabine dose reduction and dose interruption during the study period, such as the days under the treatment, the number of outpatient medications, capecitabine treatment type, and initial dosage.

It is not surprising that patients in the two samples (EHR and pilot PRO study) differed in their clinical characteristics. As a combined data set, the final sample was heterogeneous regarding cancer diagnoses. That is, the PRO sample only included patients with gastrointestinal (GI) cancer, while the EHR sample included both patients with GI cancer and patients with breast cancer. It was expected that the combined dataset would cover all cancer types that capecitabine has been indicated for. The complement of the EHR sample increased the overall sample size and balanced the combined set to be more likely to represent the patient population taking capecitabine in clinical practice. As noted in the analysis, patients from the pilot PRO study were more likely to report diarrhea, constipation, fatigue, and sleep difficulties. With the diagnosis of GI cancer, these patients might have already experienced diarrhea and constipation due to the disease. Taking capecitabine can make their experience of diarrhea and constipation worse. Regarding the less severe fatigue and sleep difficulties from the EHR sample, a potential explanation is patients may be less likely to report their subjective symptoms, such as fatigue, to clinicians during clinical visits [17], or self-reported subjective symptoms are less likely to be documented in the EHR notes [18]. Previous studies have indicated that clinicians are likely to underestimate the severity of the subjective symptoms and sometimes overlook the patient’s self-report [19, 20]. This combined secondary analysis suggests that the integration of PRO data into EHRs can be a good way to fully understand patients’ experience of treatment side effects, which is aligned with the literature that promotes EHR-integrated PROs to support high-quality patient-centered cancer care [21, 22].

The common side effects of capecitabine identified from clinical notes were like those self-reported by patients in the pilot study and those in the SIDER database [13], including fatigue, diarrhea, nausea, constipation, HFS, sleep difficulties, mouth sores, and vomiting. Compared to the literature, the patient sample in this study experienced higher incidences of fatigue (69% vs 42%), constipation (35% vs 14%), and sleep difficulties (25% vs 7%), and lower incidences of HFS (30% vs 54%) [13]. It is possible that patients sampled from routine care (documented in EHR clinical notes) may have a different level of side effect experiences compared to those in clinical trials as those trials often have strict inclusion and exclusion criteria for participation which may affect the documented occurrence of side effects [18, 23]. Regarding the lower incidence of HFS identified in the combined sample, as HFS is a well-known common and significant side effect of capecitabine, clinicians may have adopted certain strategies to control the development of HFS in clinical practice, such as more intensive assessment and management of early signs and symptoms of HFS, e.g., having dose reductions or dose interruptions [24, 25], as indicated in this analysis that the severity of HFS was significantly associated with dose reduction.

As expected, age was found to be associated with the severity of several side effects of capecitabine [26]. Specifically, this study demonstrated that older adults were vulnerable to severe fatigue and HFS of capecitabine treatment even when having other socio-demographic and clinical factors controlled in the models (see Table 3). Fatigue is one of the most prevalent cancer treatment-induced side effects [27], and older adult patients are often more susceptible to fatigue due to declined physical function [28]. The percentage of older adults who reported moderate-severe fatigue in this study (about 40%) was higher than that reported in the literature (about 25%) [29]. However, the number of older adults who rated their HFS as severe or extremely severe in this study (7%) was lower than those reported in a previous randomized controlled trial or a chart review study (21%) [30, 31]. Such differences may be because of proactive HFS monitoring and management strategies in clinical practice or the small sample of older adults (n = 43, 44%) with a limited representation. Both fatigue and HFS are OAA-related common side effects and are difficult to manage, especially in older adult patients, which can significantly impact their quality of life and may cause impairment of function [32]. Before developing any supportive programs to empower and engage older adults in side effect self-management, it is good to understand their side effect self-reporting behavior patterns. Older adults with cancer tend to under-report their symptom experiences [33]. It is uncertain whether they have strong recovery potentials due to resilience [34] or become more tolerant to side effects due to the response shift of their side effect experiences along the time [35, 36]. Capturing individual dynamic responses to OAA treatment is critical for the development of personalized interventions [34, 36]. Therefore, it is important to assess older adults’ OAA treatment tolerability in a timely fashion and monitor for early signs and symptoms of toxicities from home [37], to intervene early to prevent the development of severe side effects and improve patient safety and health outcomes [16, 38].

The current literature implies that the severity of side effects of OAAs may positively correlate with the number of outpatient medications and the number of comorbidities [9]. Concurrent medications may contribute to patients’ experiences of severe side effects of OAAs due to drug interactions or combined drug effects. It is not surprising that this study found that the number of outpatient medications was significantly associated with patients’ experience of more severe fatigue of capecitabine. Older adults in this study were found to be taking significantly more outpatient medications than younger adults, correspondingly, they presented with more fatigue. Surprisingly, older adults in this study had a smaller number of comorbidities than younger adults, which may be because of an incomplete list of comorbidities in older adults’ medication records. This may explain why there was not an association between the number of comorbidities and severe fatigue or HFS. As one of the limitations, the study did not track the specific type of comorbidities and type of concurrent medications, thus it is unclear whether specific types of comorbidities or medications may significantly interfere with older adults’ tolerance to OAA treatment, which can be further explored in future studies.

Co-occurring side effects are common in patients receiving chemotherapy [39]. Interestingly, this study revealed different patterns of co-occurring side effects of capecitabine between older and younger adults. While the severity of many side effects was found to be correlated with each other among younger adults, this was not the case in the older adult sample. For example, the severity of nausea and vomiting in younger adults was highly correlated, however, the severity of nausea in older adults was not significantly correlated with their vomiting. This may be because older adult patients often have a lower risk of chemotherapy-induced nausea and vomiting than younger adults [40]. HFS is another unique example, where there were significant correlations in the older population but not the younger sample. By contrast, diarrhea did not present any correlations with other side effects in older adults but was significantly correlated in the younger population. These different symptom cluster patterns between younger and older adults have not been reported in the literature before. As the finding of co-occurring side effects of OAA can be useful in guiding targeted side effect management interventions for older adults in the future, these findings should be highlighted and further validated by more studies with a large sample size to improve the generalizability.

While the focus of this secondary analysis was on understanding older adults’ experience of toxicities of capecitabine treatment, the exploration of the occurrence of dose reduction and dose interruption among them can improve the understanding, as dose reductions and temporary dose interruptions are commonly used as clinical strategies for OAA-related toxicities management [41]. This study indicated the proportion of older adults who experienced dose reductions or dose interruptions was higher than that of younger adults during capecitabine treatment, however, such differences were not statistically significant. The limited sample size can be one of the potential explanations for the non-significance. One previous study of patients receiving infusion chemotherapies (oxaliplatin plus fluoropyrimidines) instead of oral chemotherapy (capecitabine) [42] reported significantly more older adult patients compared to younger participants experiencing dose reductions and treatment interruptions. The literature also suggests that older adult patients have higher risks for dose reductions while taking capecitabine [43]. One study focused on capecitabine monotherapy for colorectal cancer has reported that dose reductions and dose interruptions occurred in 17–24% of patients who experienced HFS [7]. This seems to be aligned with our results that the severity of HFS was significantly associated with dose reductions. However, the severity of HFS was not significantly associated with dose interruptions, although patients with dose interruptions tended to have more severe HFS. A potential limitation of this combined secondary analysis is the presence or absence of dose reduction and dose interruption was extracted from EHR documentation, which means those patient-initiated dose changes might not be recorded if patients did not report those to the clinicians. Furthermore, the total number of occurrences of dose changes throughout the whole treatment course for each participant was difficult to extract, as some patients might initiate the treatment a long time ago or continue the treatment beyond the end of the study phase. Future prospective studies can be conducted to follow up with patients along the time and explore the trajectories of treatment plan changes and associated contexts.

Dose reduction was associated with a few demographic and clinical characteristics in this study. For example, those with breast cancer were more likely to have dose reductions than other types of cancers, which may explain why females were found to be more likely to have dose reductions than males. One previous study observed greater capecitabine side-effect severity in female patients [44]. Females might have experienced some severe side effects that led to their dose reduction. Finally, since capecitabine dose reductions can be a toxicity management strategy, it may explain why patients on capecitabine monotherapy were more likely to experience dose reductions than those on the combination of capecitabine with other chemotherapy.

### Limitations

A couple of limitations of this study have been addressed above, such as a small sample size of older adults included and using a binary measure of dose reduction and dose interruption. There are also several other potential limitations for discussions. First, as the patient sample was a combination of a previous pilot study and clinical notes review, unequal sample characteristics were present between the two data sets. For example, the pilot study only recruited patients with GI cancers, which may lead to an over-representation of patients with pancreatic cancer in the combined sample. Second, our study utilized clinical notes from patient EHRs which are known to occasionally have problems of data inaccuracy or inconsistency [45]. Annotating and coding the severity of side effects might also generate mild inconsistencies due to ambiguous descriptions in the notes. Our team of annotators used the NCI CTCAE as a reference to support the mapping of the description of side-effect severity to numerical grades, which might help keep the consistency of coding. Lastly, compared to the pilot study that collected patients’ self-reported side effects via personal phone calls, the review of the clinical notes could only obtain information that was documented by clinicians. As some patients might not report their experienced side effects for various reasons, such as fear of treatment discontinuation, this might have hindered our retrieval of representative side-effect severity data from the clinical notes.

## Conclusions

In conclusion, our combined secondary data analysis identified that older adults were more likely to experience severe fatigue and HFS during capecitabine treatment. Potential factors, such as sex, daily dose, the number of outpatient medications were associated with older adult patients’ tolerance to capecitabine treatments. Although dose reduction and dose interruption among older adult patient were not statistically significantly different from those in younger adults, the severity of HFS was associated with the presence of dose reduction. Additionally, patterns of co-occurring side effects of capecitabine significantly differed between older and younger patients. These findings can be further validated to guide the development of optimal capecitabine treatment plans and personalized toxicity monitoring and management. As capecitabine was used as an example of OAAs, findings from this study may be used to guide the understanding of older adult patients’ tolerance of other OAA treatments. More research studies with larger sample sizes are needed to help determine more details about OAA treatment responses and tolerances among older adults to provide a better quality of care for this understudied population.

## Data Availability

The datasets used and/or analyzed during the current study are available from the corresponding author on reasonable request.

## Abbreviations

OAAs: Oral anticancer agents
HFS: Hand-foot syndrome
EHR: Electronic health records
CTCAE: Common Terminology for Adverse Effects
